# Supplementary material for: The Ypk1 protein kinase signaling pathway is rewired and not essential for viability in Candida albicans
Source: PLoS Genet. 2023 Aug 10;19(8):e1010890. doi: 10.1371/journal.pgen.1010890 (PMC10443862; doi:10.1371/journal.pgen.1010890)
Supplement: S1 Fig — (PDF) [file pgen.1010890.s001.pdf]

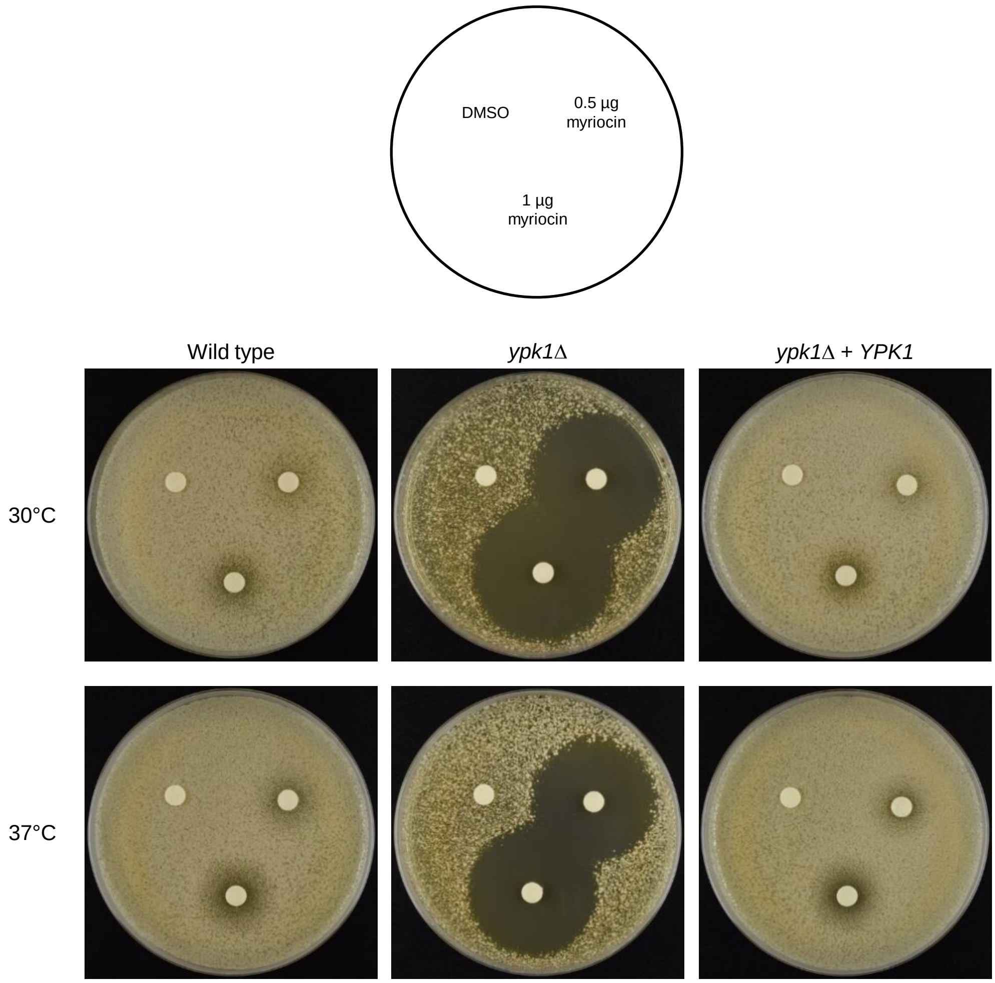

Fig S1. Confirmation of the myriocin hypersensitivity of *ypk1*Δ mutants by a disk diffusion assay. Overnight cultures of the wild-type strain SC5314, *ypk1*Δ mutants, and complemented strains were diluted to an OD_{600} of 0.2 and 200 µl of the cell suspensions was plated on YPD agar plates. Disks containing 0.5 µg myriocin, 1 µg myriocin, or the solvent control (DMSO) were placed as indicated and the plates incubated for 48 h at 30°C or 37°C. Both independently generated series of mutants behaved identically and only one of them is shown.
