# Supplementary material for: The Ypk1 protein kinase signaling pathway is rewired and not essential for viability in Candida albicans
Source: PLoS Genet. 2023 Aug 10;19(8):e1010890. doi: 10.1371/journal.pgen.1010890 (PMC10443862; doi:10.1371/journal.pgen.1010890)
Supplement: S2 Fig — (PDF) [file pgen.1010890.s002.pdf]

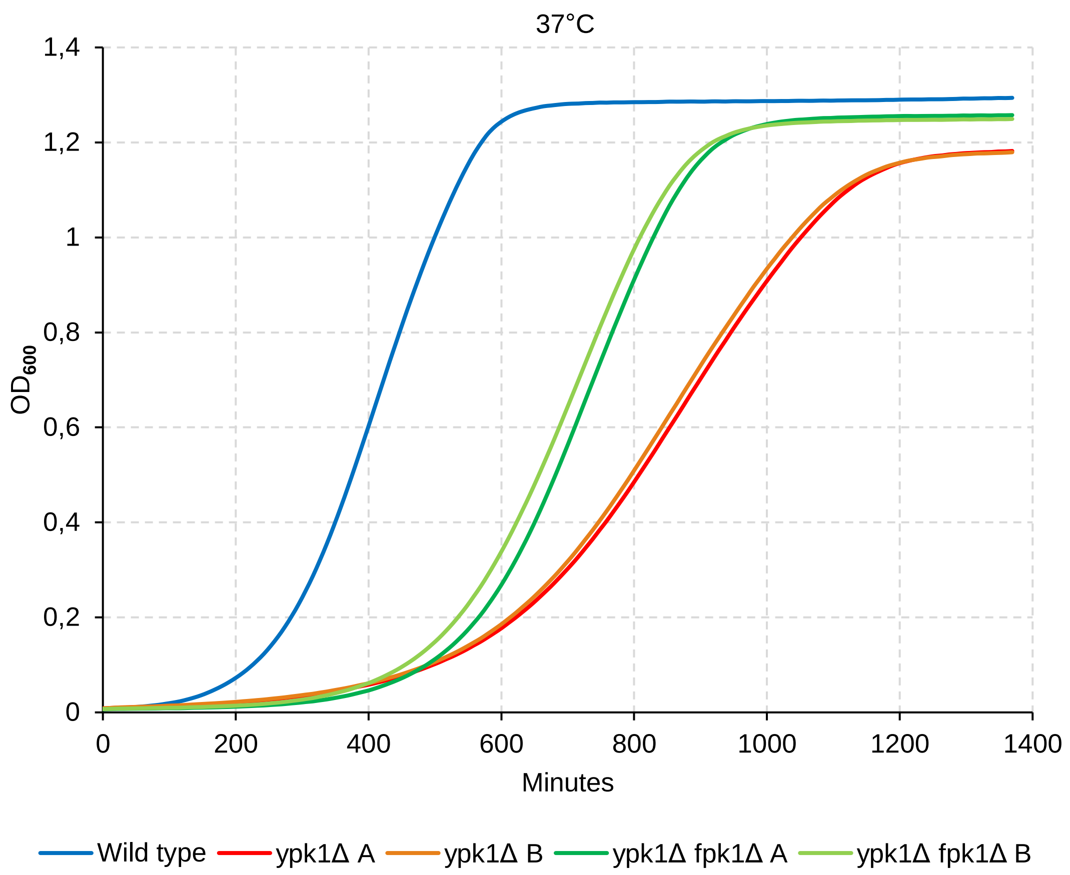

	Doubling time mean (min)	SD
WT	48.7	0.3
*ypk1*Δ A	96.0	0.9
*ypk1*Δ B	94.1	3.6
*ypk1*Δ *fpk1*Δ A	58.8	1.0
*ypk1*Δ *fpk1*Δ B	58.1	1.1

Fig S2. Growth curves of the wild-type strain SC5314, *ypk1*Δ single mutants, and *ypk1*Δ *fpk1*Δ double mutants at 37°C. Doubling times during log phase (mean and SD from three biological replicates, each with three technical replicates) are given.
