# Supplementary material for: The Ypk1 protein kinase signaling pathway is rewired and not essential for viability in Candida albicans
Source: PLoS Genet. 2023 Aug 10;19(8):e1010890. doi: 10.1371/journal.pgen.1010890 (PMC10443862; doi:10.1371/journal.pgen.1010890)
Supplement: S3 Fig — (DOCX) [file pgen.1010890.s003.docx]

*PKH1-1*: ATGCATAAATTTAGATATTCTTTGCACCAACACTATAGCAAACGCAATTCAAGTGACAAA 60

||||||||||||||||||||||||||||||||||||||||||||||||||||||||||||

[Ca22chr1A](http://www.candidagenome.org/cgi-bin/compute/blast_clade.pl#list_Ca22chr1A_C_albicans_SC5314): ATGCATAAATTTAGATATTCTTTGCACCAACACTATAGCAAACGCAATTCAAGTGACAAA 2702967

*PKH1-1*: TCCAAAGACAGTCCAATTAGCCAAAACAGCAATGAAGAAAATGATTCGACTAAATTAAGT 120

||||||||||||||||||||||||||||||||||||||||||||||||||||||||||||

Ca22chr1A: TCCAAAGACAGTCCAATTAGCCAAAACAGCAATGAAGAAAATGATTCGACTAAATTAAGT 2702907

*PKH1-1*: TCAAGTAGTCTTCAAGACTTACATGATGATCTCGATGATATTTATAACAACTATACTTTA 180

||||||||||||||||||||||||||||||||||||||||||||||||||||||||||||

Ca22chr1A: TCAAGTAGTCTTCAAGACTTACATGATGATCTCGATGATATTTATAACAACTATACTTTA 2702847

*PKH1-1*: GCACAGGGTACCAATAACAACAGTGTAGATACATTGGATTCTGAAAATAATCAAGCTATA 240

||||||||||||||||||||||||||||||||||||||||||||||||||||||||||||

Ca22chr1A: GCACAGGGTACCAATAACAACAGTGTAGATACATTGGATTCTGAAAATAATCAAGCTATA 2702787

*PKH1-1*: AATAAGTTTATTGATAAACCTCCAGCAATTCATGGTATGGAACCACAACTACCGGTGATG 300

||||||||||||||||||||||||||||||||||||||||||||||||||||||||||||

Ca22chr1A: AATAAGTTTATTGATAAACCTCCAGCAATTCATGGTATGGAACCACAACTACCGGTGATG 2702727

*PKH1-1*: CACGTTTCTTCACGATTATCTTCCTTAGGTAATACCACCAATGAAACCGGTGAAAGCATC 360

||||||||||||||||||||||||||||||||||||||||||||||||||||||||||||

Ca22chr1A: CACGTTTCTTCACGATTATCTTCCTTAGGTAATACCACCAATGAAACCGGTGAAAGCATC 2702667

*PKH1-1*: GCCAAAAGTGCACCAGGAACTCCGTTATCTTCACATTCATTTGATTTCAGACCGCATCAT 420

||||||||||||||||||||||||||||||||||||||||||||||||||||||||||||

Ca22chr1A: GCCAAAAGTGCACCAGGAACTCCGTTATCTTCACATTCATTTGATTTCAGACCGCATCAT 2702607

*PKH1-1*: CCTCGTGCAGTAACAAACTCATCCCTCAATGTATTGTTAGACACCCCTAATGTCAGTTCC 480

||||||||||||||||||||||||||||||||||||||||||||||||||||||||||||

Ca22chr1A: CCTCGTGCAGTAACAAACTCATCCCTCAATGTATTGTTAGACACCCCTAATGTCAGTTCC 2702547

*PKH1-1*: GAATTCAATCATTTAGTGGATCAAACACCACCCAATGAGTCGGTAGAAAGGTTTGACGAC 540

||||||||||||||||||||||||||||||||||||||||||||||||||||||||||||

Ca22chr1A: GAATTCAATCATTTAGTGGATCAAACACCACCCAATGAGTCGGTAGAAAGGTTTGACGAC 2702487

*PKH1-1*: AGTAATAATACTGTGGACAATACAGAAGAGGAAGAAAATAATGATGATACAGACGAAATA 600

||||||||||||||||||||||||||||||||||||||||||||||||||||||||||||

Ca22chr1A: AGTAATAATACTGTGGACAATACAGAAGAGGAAGAAAATAATGATGATACAGACGAAATA 2702427

*PKH1-1*: CCAAAATCCGAAACATTGAAACAAAACGAGGAGAATTGGGAAAAAAAGGGTGCTGCAGTT 660

||||||||||||||||||||||||||||||||||||||||||||||||||||||||||||

Ca22chr1A: CCAAAATCCGAAACATTGAAACAAAACGAGGAGAATTGGGAAAAAAAGGGTGCTGCAGTT 2702367

*PKH1-1*: AAAACTATCAAGACTATGGATGGAGAAATGAAAACTATTCGGCGAAATGTTACTGATTTC 720

||||||||||||||||||||||||||||||||||||||||||||||||||||||||||||

Ca22chr1A: AAAACTATCAAGACTATGGATGGAGAAATGAAAACTATTCGGCGAAATGTTACTGATTTC 2702307

*PKH1-1*: AAATTTGGTAAAGAATTGGGTGAAGGTTCATATTCCACGGTGATTTTAGCCACTGATAAG 780

||||||||||||||||||||||||||||||||||||||||||||||||||||||||||||

Ca22chr1A: AAATTTGGTAAAGAATTGGGTGAAGGTTCATATTCCACGGTGATTTTAGCCACTGATAAG 2702247

*PKH1-1*: ATTACTGGTAAACAATATGCTGTAAAAGTACTTGATAAGCGACATATTATAAAAGAAAAG 840

||||||||||||||||||||||||||||||||||||||||||||||||||||||||||||

Ca22chr1A: ATTACTGGTAAACAATATGCTGTAAAAGTACTTGATAAGCGACATATTATAAAAGAAAAG 2702187

*PKH1-1*: AAAGTCAAGTATGTCAATATAGAAAAACATGCATTGAATCGATTAAGTAATAGATTAGGG 900

||||||||||||||||||||||||||||||||||||||||||||||||||||||||||||

Ca22chr1A: AAAGTCAAGTATGTCAATATAGAAAAACATGCATTGAATCGATTAAGTAATAGATTAGGG 2702127

*PKH1-1*: GTTATTTCATTATATTTCACCTTCCAGGATAAAGATTCGCTTTATTTTGTTTTGGATTAT 960

||||||||||||||||||||||||||||||||||||||||||||||||||||||||||||

Ca22chr1A: GTTATTTCATTATATTTCACCTTCCAGGATAAAGATTCGCTTTATTTTGTTTTGGATTAT 2702067

*PKH1-1*: GCTTCAAATGGTGAATTATTGACATTGATCAAGAGATACAATACTTTAAATGAGGAATGT 1020

||||||||||||||||||||||||||||||||||||||||||||||||||||||||||||

Ca22chr1A: GCTTCAAATGGTGAATTATTGACATTGATCAAGAGATACAATACTTTAAATGAGGAATGT 2702007

*PKH1-1*: ACTAGACATTTTGGTGCACAAATATTAGATGCTATTAAATATATGCATGATAATGGTGTT 1080

||||||||||||||||||||||||||||||||||||||||||||||||||||||||||||

Ca22chr1A: ACTAGACATTTTGGTGCACAAATATTAGATGCTATTAAATATATGCATGATAATGGTGTT 2701947

*PKH1-1*: ATACATCGAGACCTAAAACCAGAGAATATATTATTAGATGACAAAATGAGAATTCAAATT 1140

||||||||||||||||||||||||||||||||||||||||||||||||||||||||||||

Ca22chr1A: ATACATCGAGACCTAAAACCAGAGAATATATTATTAGATGACAAAATGAGAATTCAAATT 2701887

*PKH1-1*: ACAGATTTTGGTACTGCAAGATTATTAGAGAAAAAGAATGATGAAAGTGAAGAATACCCA 1200

||||||||||||||||||||||||||||||||||||||||||||||||||||||||||||

Ca22chr1A: ACAGATTTTGGTACTGCAAGATTATTAGAGAAAAAGAATGATGAAAGTGAAGAATACCCA 2701827

*PKH1-1*: GTGGATGTAAGAGCAAAATCATTTGTTGGAACCGCTGAATATGTATCCCCTGAATTATTA 1260

||||||||||||||||||||||||||||||||||||||||||||||||||||||||||||

Ca22chr1A: GTGGATGTAAGAGCAAAATCATTTGTTGGAACCGCTGAATATGTATCCCCTGAATTATTA 2701767

*PKH1-1*: GAAAATAAGTATTGTGGTAAACCTGGAGATGTTTGGGCTTTTGGTTGCATCATATATCAA 1320

||||||||||||||||||||||||||||||||||||||||||||||||||||||||||||

Ca22chr1A: GAAAATAAGTATTGTGGTAAACCTGGAGATGTTTGGGCTTTTGGTTGCATCATATATCAA 2701707

*PKH1-1*: ATGATTGCTGGGAAACCACCATTTAAGGCAACTAATGAATATTTAACGTTTCAAAAAATT 1380

||||||||||||||||||||||||||||||||||||||||||||||||||||||||||||

Ca22chr1A: ATGATTGCTGGGAAACCACCATTTAAGGCAACTAATGAATATTTAACGTTTCAAAAAATT 2701647

*PKH1-1*: ACGAAATTGCAATTTGCGTTTAGTGCAGGATTCCCTACAATTATTAGAGATTTAATAAAG 1440

||||||||||||||||||||||||||||||||||||||||||||||||||||||||||||

Ca22chr1A: ACGAAATTGCAATTTGCGTTTAGTGCAGGATTCCCTACAATTATTAGAGATTTAATAAAG 2701587

*PKH1-1*: AAGATTCTTGTGTTGCAACCTTCACGACGTGCCACCATTCCAGAAATACAAAAACATTAC 1500

||||||||||||||||||||||||||||||||||||||||||||||||||||||||||||

Ca22chr1A: AAGATTCTTGTGTTGCAACCTTCACGACGTGCCACCATTCCAGAAATACAAAAACATTAC 2701527

*PKH1-1*: TTTTTCCAATCGGTCGACTTTAAAGATTTTGATCTGATTTGGTTGTCTGATCCTCCTGAA 1560

||||||||||||||||||||||||||||||||||||||||||||||||||||||||||||

Ca22chr1A: TTTTTCCAATCGGTCGACTTTAAAGATTTTGATCTGATTTGGTTGTCTGATCCTCCTGAA 2701467

*PKH1-1*: ATAGGACCTTATAAAATGACAGCAAAATCCATGATGAAAGTACCGGAATTGAATAAGGCA 1620

||||||||||||||||||||||||||||||||||||||||||||||||||||||||||||

Ca22chr1A: ATAGGACCTTATAAAATGACAGCAAAATCCATGATGAAAGTACCGGAATTGAATAAGGCA 2701407

*PKH1-1*: CCTATAACCACAGTCATTAAGAAGAATGTGAAGAAATCCACAAACTCAAATTCAAATACC 1680

||||||||||||||||||||||||||||||||||||||||||||||||||||||||||||

Ca22chr1A: CCTATAACCACAGTCATTAAGAAGAATGTGAAGAAATCCACAAACTCAAATTCAAATACC 2701347

*PKH1-1*: AACAATGTCGCCACTGCTGTTGGTGGTAGTAGTAGTAACGGACATAAAGGGTCATCACCG 1740

||||||||||||||||||||||||||||||||||||||||||||||||||||||||||||

Ca22chr1A: AACAATGTCGCCACTGCTGTTGGTGGTAGTAGTAGTAACGGACATAAAGGGTCATCACCG 2701287

*PKH1-1*: ACTCCTGAGAAAGAGCCGAGCCCAGCTACTATTAATAACAAGTCCACAGAAAAAGTTAGT 1800

||||||||||||||||||||||||||||||||||||||||||||||||||||||||||||

Ca22chr1A: ACTCCTGAGAAAGAGCCGAGCCCAGCTACTATTAATAACAAGTCCACAGAAAAAGTTAGT 2701227

*PKH1-1*: GCCGCTAGTGTAGCTGCATATGTTTTAAACAAACCAGCTACAAACCAAAATTCCAGTACA 1860

||||||||||||||||||||||||||||||||||||||||||||||||||||||||||||

Ca22chr1A: GCCGCTAGTGTAGCTGCATATGTTTTAAACAAACCAGCTACAAACCAAAATTCCAGTACA 2701167

*PKH1-1*: TCCGAGGATTCATCTAAGCGTAGCAGCAACTCCAATGAAACTCGCAAACTTTCATATTCA 1920

||||||||||||||||||||||||||||||||||||||||||||||||||||||||||||

Ca22chr1A: TCCGAGGATTCATCTAAGCGTAGCAGCAACTCCAATGAAACTCGCAAACTTTCATATTCA 2701107

*PKH1-1*: CAACAGGATTATATTCCGGGAACAAATATTTTACGTCCACAGATTAGTACTAGACCGTCA 1980

||||||||||||||||||||||||||||||||||||||||||||||||||||||||||||

Ca22chr1A: CAACAGGATTATATTCCGGGAACAAATATTTTACGTCCACAGATTAGTACTAGACCGTCA 2701047

*PKH1-1*: GTAGGATCTTATGTGAAAACCACACCATCAAAGGATAGAAAAACATTAACCAAGGTCCCA 2040

||||||||||||||||||||||||||||||||||||||||||||||||||||||||||||

Ca22chr1A: GTAGGATCTTATGTGAAAACCACACCATCAAAGGATAGAAAAACATTAACCAAGGTCCCA 2700987

*PKH1-1*: CTGAATATCCATCAACAACAAGAAAAAGTGAAACCGAAAGTAATGGAAGTGAAGCCAGCA 2100

||||||||||||||||||||||||||||||||||||||||||||||||||||||||||||

Ca22chr1A: CTGAATATCCATCAACAACAAGAAAAAGTGAAACCGAAAGTAATGGAAGTGAAGCCAGCA 2700927

*PKH1-1*: ACTACATTGGAAGCAGCATGGGAACCATATTTAACCCATCCAGATGAAAGAATACTTCGT 2160

||||||||||||||||||||||||||||||||||||||||||||||||||||||||||||

Ca22chr1A: ACTACATTGGAAGCAGCATGGGAACCATATTTAACCCATCCAGATGAAAGAATACTTCGT 2700867

*PKH1-1*: ATTGGTCCAGTTATTGCTCATAAAGAACCAACAGAACCATTTGAAAAGAAGAATAAAGCA 2220

||||||||||||||||||||||||||||||||||||||||||||||||||||||||||||

Ca22chr1A: ATTGGTCCAGTTATTGCTCATAAAGAACCAACAGAACCATTTGAAAAGAAGAATAAAGCA 2700807

*PKH1-1*: TCTTTACATATATCACCTTTGGATATAAATAAAGAACAAAGAAGTAGATCCAATACTAGT 2280

||||||||||||||||||||||||||||||||||||||||||||||||||||||||||||

Ca22chr1A: TCTTTACATATATCACCTTTGGATATAAATAAAGAACAAAGAAGTAGATCCAATACTAGT 2700747

*PKH1-1*: TTACTTACACAAATTGTAAATGAAGTAAACAATAACACCAGCGAATTGAAAAAAGTGGAA 2340

||||||||||||||||||||||||||||||||||||||||||||||||||||||||||||

Ca22chr1A: TTACTTACACAAATTGTAAATGAAGTAAACAATAACACCAGCGAATTGAAAAAAGTGGAA 2700687

*PKH1-1*: AATGCTGATGAATCACTTGCCATTATTGAACCACAATATAATATGAAGAGAAGTCCAACT 2400

||||||||||||||||||||||||||||||||||||||||||||||||||||||||||||

Ca22chr1A: AATGCTGATGAATCACTTGCCATTATTGAACCACAATATAATATGAAGAGAAGTCCAACT 2700627

*PKH1-1*: TCTGATAGTAAGAAAAGTATGGATATTGAAAGATCTGCATCTACTTCTGGAAGTAGAATT 2460

||||||||||||||||||||||||||||||||||||||||||||||||||||||||||||

Ca22chr1A: TCTGATAGTAAGAAAAGTATGGATATTGAAAGATCTGCATCTACTTCTGGAAGTAGAATT 2700567

*PKH1-1*: AGTAAGAAGGCAATTTTCAAAAAATTGGGGTTTAGTCATTTAGAAAAAAATGATAGTGAA 2520

||||||||||||||||||||||||||||||||||||||||||||||||||||||||||||

Ca22chr1A: AGTAAGAAGGCAATTTTCAAAAAATTGGGGTTTAGTCATTTAGAAAAAAATGATAGTGAA 2700507

*PKH1-1*: GAATCAAATGGTCCTAGTTTAACGGAAAAACCACAAACTTGTACATTGGTTGTTACAACT 2580

||||||||||||||||||||||||||||||||||||||||||||||||||||||||||||

Ca22chr1A: GAATCAAATGGTCCTAGTTTAACGGAAAAACCACAAACTTGTACATTGGTTGTTACAACT 2700447

*PKH1-1*: CATGGTCGAGCATTACTTTTCATTAGAAATGATATAGAATCCAATTATCTTTTAATTGCT 2640

||||||||||||||||||||||||||||||||||||||||||||||||||||||||||||

Ca22chr1A: CATGGTCGAGCATTACTTTTCATTAGAAATGATATAGAATCCAATTATCTTTTAATTGCT 2700387

*PKH1-1*: GAAATCAAATTGAAATATCCATTTATTCATTTCCAAGAATTAGTTATATCACAAACTAAA 2700

||||||||||||||||||||||||||||||||||||||||||||||||||||||||||||

Ca22chr1A: GAAATCAAATTGAAATATCCATTTATTCATTTCCAAGAATTAGTTATATCACAAACTAAA 2700327

*PKH1-1*: TTTTCTAAATTAGTACCATCAGTCGGAGTATTTGTCATTAGTTCAATTGATAATTCATTA 2760

||||||||||||||||||||||||||||||||||||||||||||||||||||||||||||

Ca22chr1A: TTTTCTAAATTAGTACCATCAGTCGGAGTATTTGTCATTAGTTCAATTGATAATTCATTA 2700267

*PKH1-1*: ATTTTTGAAGTAGAAAAATTTGAAGTGAATCAATGGACTGAAGCATTAGCTAAATCTAAA 2820

||||||||||||||||||||||||||||||||||||||||||||||||||||||||||||

Ca22chr1A: ATTTTTGAAGTAGAAAAATTTGAAGTGAATCAATGGACTGAAGCATTAGCTAAATCTAAA 2700207

*PKH1-1*: TATAATGAAATT-GAAAGAGGTAAATTAGCTGCATATGAAAGTCAAGTATCATTGAAAGA 2879

|||||||||||| |||||||||||||||||||||||||||||||||||||||||||||||

Ca22chr1A: TATAATGAAATTGGAAAGAGGTAAATTAGCTGCATATGAAAGTCAAGTATCATTGAAAGA 2700147

*PKH1-1*: ACCAGTTGTGATTAAAAAACAGTCATCATCTACTTATAATACTACACGTTCATCACCAAA 2939

||||||||||| | ||||||||||||||||||||||||||||||||||||||||||||||

Ca22chr1A: ACCAGTTGTGA-TWAAAAACAGTCATCATCTACTTATAATACTACACGTTCATCACCAAA 2700088

*PKH1-1*: ACTTTTAGATGCTCCAGCATTTGATTCTTCAGTTGCAAAAACTAATAATCATAAAAAATC 2999

||||||||||||||||||||||||||||||||||||||||||||||||||||||||||||

Ca22chr1A: ACTTTTAGATGCTCCAGCATTTGATTCTTCAGTTGCAAAAACTAATAATCATAAAAAATC 2700028

*PKH1-1*: ACAGCAGCATTCTTCTCCAACAGTTAGGAAGTCATTTGAAACATCAAGTCCATCATCACA 3059

||||||||||||||||||||||||||||||||||||||||||||||||||||||||||||

Ca22chr1A: ACAGCAGCATTCTTCTCCAACAGTTAGGAAGTCATTTGAAACATCAAGTCCATCATCACA 2699968

*PKH1-1*: TTCACCGCGTACAGCTGCCACAAATATGTTTAAAATGAAATTACAATCAGGTACTCCAAA 3119

||||||||||||||||||||||||||||||||||||||||||||||||||||||||||||

Ca22chr1A: TTCACCGCGTACAGCTGCCACAAATATGTTTAAAATGAAATTACAATCAGGTACTCCAAA 2699908

*PKH1-1*: GAGGAAACCACCACCTCCAGTGAGTCCACCTCAAGAATTAAATTCTCATACTGGATTACC 3179

||||||||||||||||||||||||||||||||||||||||||||||||||||||||||||

Ca22chr1A: GAGGAAACCACCACCTCCAGTGAGTCCACCTCAAGAATTAAATTCTCATACTGGATTACC 2699848

*PKH1-1*: TAGAAGATCAACTGAAAATGGAACTTTACATGCTGCTCAATTGGCAGTTTCTCAAGTTTC 3239

||||||||||||||||||||||||||||||||||||||||||||||||||||||||||||

Ca22chr1A: TAGAAGATCAACTGAAAATGGAACTTTACATGCTGCTCAATTGGCAGTTTCTCAAGTTTC 2699788

*PKH1-1*: CCCGAAAGGATATTCAAATAATAGACGATCAAGTTTTACAAAAGAAGATGGAAGCAAAAT 3299

||||||||||||||||||||||||||||||||||||||||||||||||||||||||||||

Ca22chr1A: CCCGAAAGGATATTCAAATAATAGACGATCAAGTTTTACAAAAGAAGATGGAAGCAAAAT 2699728

*PKH1-1*: TGATCCAAATAAAATGAGAAAAGCTGCCCATACTTATAATCACAGTCATAGTCATAATGG 3359

||||||||||||||||||||||||||||||||||||||||||||||||||||||||||||

Ca22chr1A: TGATCCAAATAAAATGAGAAAAGCTGCCCATACTTATAATCACAGTCATAGTCATAATGG 2699668

*PKH1-1*: TAACAACGGCAACAGTAACAGTAATAGTAATGGTAATAAAAGTGGATCTGGGGTAACTGC 3419

||||||||||||||||||||||||||||||||||||||||||||||||||||||||||||

Ca22chr1A: TAACAACGGCAACAGTAACAGTAATAGTAATGGTAATAAAAGTGGATCTGGGGTAACTGC 2699608

*PKH1-1*: ATTAAATTCTAAATTTTTAGCTAGAAGTACTAGGAAAAAATGA 3462

|||||||||||||||||||||||||||||||||||||||||||

Ca22chr1A: ATTAAATTCTAAATTTTTAGCTAGAAGTACTAGGAAAAAATGA 2699565

*PKH1-2*: ATGCATAAATTTAGATATTCTTTGCACCAACACTATAGCAAACGCAATTCAAGTGACAAA 60

||||||||||||||||||||||||||||||||||||||||||||||||||||||||||||

Ca22chr1B: ATGCATAAATTTAGATATTCTTTGCACCAACACTATAGCAAACGCAATTCAAGTGACAAA 2703007

*PKH1-2*: TCCAAAGACAGTCCAATTAGCCAAAACAGCAATGAAGAAAATGATTCGACTAAATTAAGT 120

||||||||||||||||||||||||||||||||||||||||||||||||||||||||||||

Ca22chr1B: TCCAAAGACAGTCCAATTAGCCAAAACAGCAATGAAGAAAATGATTCGACTAAATTAAGT 2702947

*PKH1-2*: TCAAGTAGTCTTCAAGACTTACATGATGATCTCGATGATATTTATAACAACTATACTTTA 180

||||||||||||||||||||||||||||||||||||||||||||||||||||||||||||

Ca22chr1B: TCAAGTAGTCTTCAAGACTTACATGATGATCTCGATGATATTTATAACAACTATACTTTA 2702887

*PKH1-2*: GCACAGGGTACCAATAACAACAGTGTAGATACATTGGATTCTGAAAATAATCAAGCTATA 240

||||||||||||||||||||||||||||||||||||||||||||||||||||||||||||

Ca22chr1B: GCACAGGGTACCAATAACAACAGTGTAGATACATTGGATTCTGAAAATAATCAAGCTATA 2702827

*PKH1-2*: AATAAGTTTATTGATAAACCTCCAGCAATTCATGGTATGGAACCACAACTACCGGTGATG 300

||||||||||||||||||||||||||||||||||||||||||||||||||||||||||||

Ca22chr1B: AATAAGTTTATTGATAAACCTCCAGCAATTCATGGTATGGAACCACAACTACCGGTGATG 2702767

*PKH1-2*: CACGTTTCTTCACGATTATCTTCCTTAGGTAATACCACCAATGAAACCGGTGAAAGCATC 360

||||||||||||||||||||||||||||||||||||||||||||||||||||||||||||

Ca22chr1B: CACGTTTCTTCACGATTATCTTCCTTAGGTAATACCACCAATGAAACCGGTGAAAGCATC 2702707

*PKH1-2*: GCCAAAAGTGCACCAGGAACTCCGTTATCTTCACATTCATTTGATTTCAGACCGCATCAT 420

||||||||||||||||||||||||||||||||||||||||||||||||||||||||||||

Ca22chr1B: GCCAAAAGTGCACCAGGAACTCCGTTATCTTCACATTCATTTGATTTCAGACCGCATCAT 2702647

*PKH1-2*: CCTCGTGCAGTAACAAACTCATCCCTCAATGTATTGTTAGACACCCCTAATGTCAGTTCC 480

||||||||||||||||||||||||||||||||||||||||||||||||||||||||||||

Ca22chr1B: CCTCGTGCAGTAACAAACTCATCCCTCAATGTATTGTTAGACACCCCTAATGTCAGTTCC 2702587

*PKH1-2*: GAATTCAATCATTTAGTGGATCAAACACCACCCAATGAGTCGGTAGAAAGGTTTGACGAC 540

||||||||||||||||||||||||||||||||||||||||||||||||||||||||||||

Ca22chr1B: GAATTCAATCATTTAGTGGATCAAACACCACCCAATGAGTCGGTAGAAAGGTTTGACGAC 2702527

*PKH1-2*: AGTAATAATACTGTGGACAATACAGAAGAGGAAGAAAATAATGATGATACAGACGAAATA 600

||||||||||||||||||||||||||||||||||||||||||||||||||||||||||||

Ca22chr1B: AGTAATAATACTGTGGACAATACAGAAGAGGAAGAAAATAATGATGATACAGACGAAATA 2702467

*PKH1-2*: CCAAAATCCGAAACATTGAAACAAAACGAGGAGAATTGGGAAAAAAAGGGTGCTGCAGTT 660

||||||||||||||||||||||||||||||||||||||||||||||||||||||||||||

Ca22chr1B: CCAAAATCCGAAACATTGAAACAAAACGAGGAGAATTGGGAAAAAAAGGGTGCTGCAGTT 2702407

*PKH1-2*: AAAACTATCAAGACTATGGATGGAGAAATGAAAACTATTCGGCGAAATGTTACTGATTTC 720

||||||||||||||||||||||||||||||||||||||||||||||||||||||||||||

Ca22chr1B: AAAACTATCAAGACTATGGATGGAGAAATGAAAACTATTCGGCGAAATGTTACTGATTTC 2702347

*PKH1-2*: AAATTTGGTAAAGAATTGGGTGAAGGTTCATATTCCACGGTGATTTTAGCCACTGATAAG 780

||||||||||||||||||||||||||||||||||||||||||||||||||||||||||||

Ca22chr1B: AAATTTGGTAAAGAATTGGGTGAAGGTTCATATTCCACGGTGATTTTAGCCACTGATAAG 2702287

*PKH1-2*: ATTACTGGTAAACAATATGCTGTAAAAGTACTTGATAAGCGACATATTATAAAAGAAAAG 840

||||||||||||||||||||||||||||||||||||||||||||||||||||||||||||

Ca22chr1B: ATTACTGGTAAACAATATGCTGTAAAAGTACTTGATAAGCGACATATTATAAAAGAAAAG 2702227

*PKH1-2*: AAAGTCAAGTATGTCAATATAGAAAAACATGCATTGAATCGATTAAGTAATAGATTAGGG 900

||||||||||||||||||||||||||||||||||||||||||||||||||||||||||||

Ca22chr1B: AAAGTCAAGTATGTCAATATAGAAAAACATGCATTGAATCGATTAAGTAATAGATTAGGG 2702167

*PKH1-2*: GTTATTTCATTATATTTCACCTTCCAGGATAAAGATTCGCTTTATTTTGTTTTGGATTAT 960

||||||||||||||||||||||||||||||||||||||||||||||||||||||||||||

Ca22chr1B: GTTATTTCATTATATTTCACCTTCCAGGATAAAGATTCGCTTTATTTTGTTTTGGATTAT 2702107

*PKH1-2*: GCTTCAAATGGTGAATTATTGACATTGATCAAGAGATACAATACTTTAAATGAGGAATGT 1020

||||||||||||||||||||||||||||||||||||||||||||||||||||||||||||

Ca22chr1B: GCTTCAAATGGTGAATTATTGACATTGATCAAGAGATACAATACTTTAAATGAGGAATGT 2702047

*PKH1-2*: ACTAGACATTTTGGTGCACAAATATTAGATGCTATTAAATATATGCATGATAATGGTGTT 1080

||||||||||||||||||||||||||||||||||||||||||||||||||||||||||||

Ca22chr1B: ACTAGACATTTTGGTGCACAAATATTAGATGCTATTAAATATATGCATGATAATGGTGTT 2701987

*PKH1-2*: ATACATCGAGACCTAAAACCAGAGAATATATTATTAGATGACAAAATGAGAATTCAAATT 1140

||||||||||||||||||||||||||||||||||||||||||||||||||||||||||||

Ca22chr1B: ATACATCGAGACCTAAAACCAGAGAATATATTATTAGATGACAAAATGAGAATTCAAATT 2701927

*PKH1-2*: ACAGATTTTGGTACTGCAAGATTATTAGAGAAAAAGAATGATGAAAGTGAAGAATACCCA 1200

||||||||||||||||||||||||||||||||||||||||||||||||||||||||||||

Ca22chr1B: ACAGATTTTGGTACTGCAAGATTATTAGAGAAAAAGAATGATGAAAGTGAAGAATACCCA 2701867

*PKH1-2*: GTGGATGTAAGAGCAAAATCATTTGTTGGAACCGCTGAATATGTATCCCCTGAATTATTA 1260

||||||||||||||||||||||||||||||||||||||||||||||||||||||||||||

Ca22chr1B: GTGGATGTAAGAGCAAAATCATTTGTTGGAACCGCTGAATATGTATCCCCTGAATTATTA 2701807

*PKH1-2*: GAAAATAAGTATTGTGGTAAACCTGGAGATATTTGGGCTTTTGGTTGCATCATATATCAA 1320

||||||||||||||||||||||||||||||||||||||||||||||||||||||||||||

Ca22chr1B: GAAAATAAGTATTGTGGTAAACCTGGAGATATTTGGGCTTTTGGTTGCATCATATATCAA 2701747

*PKH1-2*: ATGATTGCTGGGAAACCACCATTTAAGGCAACTAATGAATATTTAACGTTTCAAAAAATC 1380

||||||||||||||||||||||||||||||||||||||||||||||||||||||||||||

Ca22chr1B: ATGATTGCTGGGAAACCACCATTTAAGGCAACTAATGAATATTTAACGTTTCAAAAAATC 2701687

*PKH1-2*: ACGAAATTGCAATTTGCGTTTAGTGCAGGATTCCCTACAATTATTAGAGATTTAATAAAG 1440

||||||||||||||||||||||||||||||||||||||||||||||||||||||||||||

Ca22chr1B: ACGAAATTGCAATTTGCGTTTAGTGCAGGATTCCCTACAATTATTAGAGATTTAATAAAG 2701627

*PKH1-2*: AAGATTCTTGTGTTGCAACCATCACGACGTGCCACCATTCCAGAAATACAAAAACATTAC 1500

||||||||||||||||||||||||||||||||||||||||||||||||||||||||||||

Ca22chr1B: AAGATTCTTGTGTTGCAACCATCACGACGTGCCACCATTCCAGAAATACAAAAACATTAC 2701567

*PKH1-2*: TTTTTCCAATCGGTTGACTTTAAAGATTTTGATCTGATTTGGTTATCTGATCCTCCAGAA 1560

||||||||||||||||||||||||||||||||||||||||||||||||||||||||||||

Ca22chr1B: TTTTTCCAATCGGTTGACTTTAAAGATTTTGATCTGATTTGGTTATCTGATCCTCCAGAA 2701507

*PKH1-2*: ATAGGACCTTATAAAATGACAGCAAAATCCATGATGAAAGTACCGGAATTGAATAAGGCA 1620

||||||||||||||||||||||||||||||||||||||||||||||||||||||||||||

Ca22chr1B: ATAGGACCTTATAAAATGACAGCAAAATCCATGATGAAAGTACCGGAATTGAATAAGGCA 2701447

*PKH1-2*: CCTGTAACCACAGTTATTAAGAAGAATGTCAAGAAATCCACAAACTCAAATTCAAATACC 1680

||||||||||||||||||||||||||||||||||||||||||||||||||||||||||||

Ca22chr1B: CCTGTAACCACAGTTATTAAGAAGAATGTCAAGAAATCCACAAACTCAAATTCAAATACC 2701387

*PKH1-2*: AACAATGTCACCACTGCTGTTGGTGGTGGTAGTAGTAACGGACATAAAGGGTCATCACCG 1740

||||||||||||||||||||||||||||||||||||||||||||||||||||||||||||

Ca22chr1B: AACAATGTCACCACTGCTGTTGGTGGTGGTAGTAGTAACGGACATAAAGGGTCATCACCG 2701327

*PKH1-2*: ACTCCTGAGAAAGAGCCGAGTCCAGCTACTATTAATAACAAGTCCACAGAAAAAGTTAGT 1800

||||||||||||||||||||||||||||||||||||||||||||||||||||||||||||

Ca22chr1B: ACTCCTGAGAAAGAGCCGAGTCCAGCTACTATTAATAACAAGTCCACAGAAAAAGTTAGT 2701267

*PKH1-2*: GCCGCTAGTGTAGCTGCATATGTTTTAAACAAACCAGCTACAAACCAAAATTCCAGTACA 1860

||||||||||||||||||||||||||||||||||||||||||||||||||||||||||||

Ca22chr1B: GCCGCTAGTGTAGCTGCATATGTTTTAAACAAACCAGCTACAAACCAAAATTCCAGTACA 2701207

*PKH1-2*: TCCGAGGATTCATCTAAGCGTAGCAGCAACTCCAATGAAACTCGCAAACTTTCATATTCA 1920

||||||||||||||||||||||||||||||||||||||||||||||||||||||||||||

Ca22chr1B: TCCGAGGATTCATCTAAGCGTAGCAGCAACTCCAATGAAACTCGCAAACTTTCATATTCA 2701147

*PKH1-2*: CAACAGGATTATATTCCGGGAACAAATATTTTACGTCCACAGATTAGTACTAGACCGTCA 1980

||||||||||||||||||||||||||||||||||||||||||||||||||||||||||||

Ca22chr1B: CAACAGGATTATATTCCGGGAACAAATATTTTACGTCCACAGATTAGTACTAGACCGTCA 2701087

*PKH1-2*: GTAGGATCTTATGTGAAAACCACACCATCAAAGGATAGAAAAACATTAACCAAGGTCCCA 2040

||||||||||||||||||||||||||||||||||||||||||||||||||||||||||||

Ca22chr1B: GTAGGATCTTATGTGAAAACCACACCATCAAAGGATAGAAAAACATTAACCAAGGTCCCA 2701027

*PKH1-2*: CTGAATATCCATCAACAACAAGAAAAAGTGAAACCGAAAGTAATGGAAGTGAAGCCAGCA 2100

||||||||||||||||||||||||||||||||||||||||||||||||||||||||||||

Ca22chr1B: CTGAATATCCATCAACAACAAGAAAAAGTGAAACCGAAAGTAATGGAAGTGAAGCCAGCA 2700967

*PKH1-2*: ACTACATTGGAAGCAGCATGGGAACCATATTTAACCCATCCAGATGAAAGAATACTTCGT 2160

||||||||||||||||||||||||||||||||||||||||||||||||||||||||||||

Ca22chr1B: ACTACATTGGAAGCAGCATGGGAACCATATTTAACCCATCCAGATGAAAGAATACTTCGT 2700907

*PKH1-2*: ATTGGCCCAGTTATTGCTCATAAGGAACCAACAGAACCATTTGAAAAGAAGAATAAAGCG 2220

||||||||||||||||||||||||||||||||||||||||||||||||||||||||||||

Ca22chr1B: ATTGGCCCAGTTATTGCTCATAAGGAACCAACAGAACCATTTGAAAAGAAGAATAAAGCG 2700847

*PKH1-2*: TCTTTACATATATCACCTTTGGATATAAATAAAGAACAAAGAAGTAGATCCAATACTAGT 2280

||||||||||||||||||||||||||||||||||||||||||||||||||||||||||||

Ca22chr1B: TCTTTACATATATCACCTTTGGATATAAATAAAGAACAAAGAAGTAGATCCAATACTAGT 2700787

*PKH1-2*: TTACTTACACAAATTGTAAATGAAGTAAACAATAACACCAGTGAATTGAAAAAAGTGGAA 2340

||||||||||||||||||||||||||||||||||||||||||||||||||||||||||||

Ca22chr1B: TTACTTACACAAATTGTAAATGAAGTAAACAATAACACCAGTGAATTGAAAAAAGTGGAA 2700727

*PKH1-2*: AATGCTGATGAATCACTTGCCATTATTGAACCACAATATAATATGAAGAGAAGTCCAACT 2400

||||||||||||||||||||||||||||||||||||||||||||||||||||||||||||

Ca22chr1B: AATGCTGATGAATCACTTGCCATTATTGAACCACAATATAATATGAAGAGAAGTCCAACT 2700667

*PKH1-2*: TCTGATAGTAAGAAAAGTATGGATATTGAAAGATCTGCATCTACTTCTGGAAGTAGAATT 2460

||||||||||||||||||||||||||||||||||||||||||||||||||||||||||||

Ca22chr1B: TCTGATAGTAAGAAAAGTATGGATATTGAAAGATCTGCATCTACTTCTGGAAGTAGAATT 2700607

*PKH1-2*: AGTAAAAAGGCAATTTTCAAAAAATTGGGGTTTAGTCATTTAGAAAAAAATGATACTGAA 2520

||||||||||||||||||||||||||||||||||||||||||||||||||||||||||||

Ca22chr1B: AGTAAAAAGGCAATTTTCAAAAAATTGGGGTTTAGTCATTTAGAAAAAAATGATACTGAA 2700547

*PKH1-2*: GAATCGAATGGTCCTAGTTTAACGGAAAAACCTCAAACTTGTACATTGGTTGTTACAACT 2580

||||||||||||||||||||||||||||||||||||||||||||||||||||||||||||

Ca22chr1B: GAATCGAATGGTCCTAGTTTAACGGAAAAACCTCAAACTTGTACATTGGTTGTTACAACT 2700487

*PKH1-2*: CATGGTCGAGCATTACTTTTCATTAGAAATGATATAGAATCCAATTATCTTTTAATTGCT 2640

||||||||||||||||||||||||||||||||||||||||||||||||||||||||||||

Ca22chr1B: CATGGTCGAGCATTACTTTTCATTAGAAATGATATAGAATCCAATTATCTTTTAATTGCT 2700427

*PKH1-2*: GAAATCAAATTGAAATATCCATTTATTCACTTCCAAGAATTAGTCATATCACAAAATAAA 2700

||||||||||||||||||||||||||||||||||||||||||||||||||||||||||||

Ca22chr1B: GAAATCAAATTGAAATATCCATTTATTCACTTCCAAGAATTAGTCATATCACAAAATAAA 2700367

*PKH1-2*: TTTTCTAAATTAGTACCATCAGTTGGGGTGTTTGTCATTAGTTCAATTGATAATTCATTA 2760

||||||||||||||||||||||||||||||||||||||||||||||||||||||||||||

Ca22chr1B: TTTTCTAAATTAGTACCATCAGTTGGGGTGTTTGTCATTAGTTCAATTGATAATTCATTA 2700307

*PKH1-2*: ATTTTTGAAGTGGAAAAATTTGAAGTGAATCAATGGACTGAAGCATTAGCTAAATCTAAA 2820

||||||||||||||||||||||||||||||||||||||||||||||||||||||||||||

Ca22chr1B: ATTTTTGAAGTGGAAAAATTTGAAGTGAATCAATGGACTGAAGCATTAGCTAAATCTAAA 2700247

*PKH1-2*: TATAATGAAATT-GAAAGAGGTAAATTAGCTGCATATGAAAGTCAAGTATCATTGAAAGA 2879

|||||||||||| |||||||||||||||||||||||||||||||||||||||||||||||

Ca22chr1B: TATAATGAAATTGGAAAGAGGTAAATTAGCTGCATATGAAAGTCAAGTATCATTGAAAGA 2700187

*PKH1-2*: ACCAGTTGTGATTAAAAAACAGTCATCATCAACTTATAATACTACACGTTCATCACCAAA 2939

||||||||||| | ||||||||||||||||||||||||||||||||||||||||||||||

Ca22chr1B: ACCAGTTGTGA-TWAAAAACAGTCATCATCAACTTATAATACTACACGTTCATCACCAAA 2700128

*PKH1-2*: ACTTTTAGATGCTCCAGAATTTGATTCTTCTGCTGCAAAAACTAATAATCATAAAAAATC 2999

||||||||||||||||||||||||||||||||||||||||||||||||||||||||||||

Ca22chr1B: ACTTTTAGATGCTCCAGAATTTGATTCTTCTGCTGCAAAAACTAATAATCATAAAAAATC 2700068

*PKH1-2*: ACAGCAGCATTCTTCTCCGACAGTTAGGAAATCATCTGAAACATCAAGTCCATCATCACA 3059

||||||||||||||||||||||||||||||||||||||||||||||||||||||||||||

Ca22chr1B: ACAGCAGCATTCTTCTCCGACAGTTAGGAAATCATCTGAAACATCAAGTCCATCATCACA 2700008

*PKH1-2*: TTCACCACGTACAGTTGCCACAAATATGTTTAAAATGAAATTACAATCAGGTACTCCAAA 3119

||||||||||||||||||||||||||||||||||||||||||||||||||||||||||||

Ca22chr1B: TTCACCACGTACAGTTGCCACAAATATGTTTAAAATGAAATTACAATCAGGTACTCCAAA 2699948

*PKH1-2*: GAGGAAACCACCACCTCCAGTGAGTCCACCTCAAGAATTAAATTCTCATACTGGATTACC 3179

||||||||||||||||||||||||||||||||||||||||||||||||||||||||||||

Ca22chr1B: GAGGAAACCACCACCTCCAGTGAGTCCACCTCAAGAATTAAATTCTCATACTGGATTACC 2699888

*PKH1-2*: TAGAAGATCAACTGAAAATGGAACTTTACATGCTGCTCAATTGGCAGTTTCTCAAGTTTC 3239

||||||||||||||||||||||||||||||||||||||||||||||||||||||||||||

Ca22chr1B: TAGAAGATCAACTGAAAATGGAACTTTACATGCTGCTCAATTGGCAGTTTCTCAAGTTTC 2699828

*PKH1-2*: TCCGAAAGGATATTCAAATAATAGACGATCAAGTTTTACAAAAGAAGATGGAAGTAAAAT 3299

||||||||||||||||||||||||||||||||||||||||||||||||||||||||||||

Ca22chr1B: TCCGAAAGGATATTCAAATAATAGACGATCAAGTTTTACAAAAGAAGATGGAAGTAAAAT 2699768

*PKH1-2*: TGATCCAAATAAAATGAGAAAAGCTGCCCATACTTATAATCACAGTCATAGTCATAATGG 3359

||||||||||||||||||||||||||||||||||||||||||||||||||||||||||||

Ca22chr1B: TGATCCAAATAAAATGAGAAAAGCTGCCCATACTTATAATCACAGTCATAGTCATAATGG 2699708

*PKH1-2*: TAACAACGGCAACAGTAACAGTAATAGTAATGGTAATAAAAGTGGATCTGGGGTAACTGC 3419

||||||||||||||||||||||||||||||||||||||||||||||||||||||||||||

Ca22chr1B: TAACAACGGCAACAGTAACAGTAATAGTAATGGTAATAAAAGTGGATCTGGGGTAACTGC 2699648

*PKH1-2*: ATTAAATTCTAAATTTTTAGCTAGAAGTACTAGGAAAAAATGA 3462

|||||||||||||||||||||||||||||||||||||||||||

Ca22chr1B: ATTAAATTCTAAATTTTTAGCTAGAAGTACTAGGAAAAAATGA 2699605

**Fig S3.** **Comparison of the coding sequences of the cloned *PKH1‑1* and *PKH1‑2* alleles with the corresponding sequences in assembly 22 of the *C. albicans* genome sequence**. The predicted orf19.5224 and orf19.5225 in CGD are shown in green and nucleotide differences in our cloned *PKH1* alleles compared to the assembly22 sequences are highlighted in red.
