# Supplementary material for: The Ypk1 protein kinase signaling pathway is rewired and not essential for viability in Candida albicans
Source: PLoS Genet. 2023 Aug 10;19(8):e1010890. doi: 10.1371/journal.pgen.1010890 (PMC10443862; doi:10.1371/journal.pgen.1010890)
Supplement: Attachment — Submitted filename: Response to reviewers.docx [file pgen.1010890.s006.docx]

**Responses to the reviewers’ comments**

Our responses are highlighted in blue for clarity.

Reviewer #1:

This study dissects the signaling pathway components and outputs associated with Candida albicans Ypk1, a highly conserved protein kinase that governs membrane structure. The study looks both upstream and downstream of Ypk1, relying upon guidance from the Ypk1 pathway in S. cerevisiae and other eukaryotes along with some judgment calls based on homology. Major conclusions are based upon deletion mutants, site-directed mutants, analysis of protein phosphorylation on Western blots, and mutant biological phenotypes. Major novel findings include:

1. YPK1 is not essential in C. albicans, in contrast with results from (at least) two previous independent studies. The game-changer here was application of the inducible FLP-deletion strategy, which the authors pioneered and used recently to show that another previously deduced essential gene (SNF1) is not essential.

2. Upstream kinase Pkh1 has overlapping function with Pkh3, a protein kinase with no known role in Ypk1 signaling in S. cerevisiae.

3. PKH1 has a longer coding region than deduced from assembly 22; it is actually a fusion of the ORFs 19.5224 and 19.5225. (The authors' finding is consistent with the Bruno RNASeq tracks displayed at CGD.)

The manuscript itself presents the work efficiently and thoughtfully. Every now and then I found myself thinking "Oh, they might want to consider this," and then a page later I'd read the authors' thoughts about just that consideration.

One really interesting observation is that this pathway has a role in hypha formation, as presented in Figure 8. The authors use an extremely strong inducing condition, hence the defects seem to be severe. The pkh1 pkh3 mutant in particular seems to grow exclusively as yeast cells. This might be an interesting finding to pursue in future studies.

One question I had as I read the manuscript was why YPK1 is not essential in C. albicans. I would be curious to hear the authors' thoughts. Maybe the authors could speculate about this issue in a sentence or two.

The viability of *C. albicans* cells lacking Ypk1 may be explained by the fact that, despite their defects in lipid homeostasis, membrane functionality is sufficiently retained to enable survival and slow growth. This is explained in the results (lines 161-163) and discussion (lines 358-362) and also in the legend to the new Fig. 9.

My only important criticism is that the manuscript does not have as clear a focal point as it could. Part of this is a consequence of the breadth of analysis that is presented. The one suggestion that I have in this regard is to create a summary figure that compares and contrasts the Ypk1 signaling pathway in S. cerevisiae and C. albicans. Either a pair of wiring diagrams or a simple table might provide the reader with a digestible reminder of the major differences and similarities.

We have added a summarizing figure, as suggested (the new Fig. 9).

One minor criticism is that the title seems to include an internal contradiction - that the pathway is conserved yet rewired. The authors might consider deleting the term "conserved."

We have deleted the word “conserved” from the title, as suggested.

Reviewer #2: This manuscript describes the phenotypes for C. albicans mutants lacking members of the conserved protein kinase cascade (Pkh, Ypk1, Fpk1). This kinase cascade was identified in S. cerevisiae as being important for regulating aspects of membrane function and stress resistance, including sphingolipid synthesis, endocytosis, and phospholipid asymmetry. A strong point of the paper is that they used a regulatable deletion system to demonstrate that the ypk1D mutant is very slow growing (whereas it had been assumed YPK1 was essential because it could not be mutated in previous studies). The authors also discovered that, in contrast to S. cerevisiae, both Pkh1 and Pkh3 play important roles in regulating Ypk1. However, weak points of the manuscript were that the endocytosis assay was not done in a sensitive manner and several assays were only presented in a qualitative manner that lacked quantitative conclusions. Altogether, this study is significant for identifying the roles of the conserved Ypk1 kinase in a major fungal pathogen.

1 Fig. 2A. It is not clear that myriocin has a greater effect on the ypk1D mutant just from looking at this spot assay. Myriocin affects the growth of both WT and ypk1D mutants, and since the ypk1D mutant grows slower the effects appear to be more dramatic. Therefore, quantitative assays should be done.

We have repeated the assay using a lower myriocin concentration that did not inhibit growth of the wild type, but virtually abolished growth of the *ypk1*Δ mutants (see the new version of Fig. 2A, in which the previous photos have been replaced). In addition, we performed a disc diffusion assay, in which the hypersensitivity of the *ypk1*Δ mutants becomes obvious by the strongly increased inhibition zone irrespective of their slower growth. This result is shown in the new supplemental Fig. S1 (the previous Fig. S1 is now Fig. S3). The description of the results in the text has been modified accordingly (lines 142-147).

2. Fig. 2C. The experiment that is presented is not sufficient to support the conclusion that the ypk1D mutant does not have an endocytosis defect. The cells were stained with FM4-64 for a long time (30 min) and then cells were incubated for an additional 90 min. This very long incubation could easily mask even a strong endocytosis defect. In order to support the conclusion that the ypk1D mutant does not have an endocytosis defect, a shorter time of FM4-64 treatment should be used and then short times of incubation should be analyzed, such as 15 min and 30 min.

We have repeated the experiment with shorter incubation times, as suggested. The results shown in the modified Fig. 2C confirm our previous conclusion that the *ypk1*Δ mutants do not have an obvious defect in endocytosis. We thank the reviewer for pointing out the limitations of our previous experimental conditions.

3. The growth rate of the wild type and ypk1D strains should be quantified to better understand how much slower is the growth of the ypk1D mutant.

See response to next, related comment.

4. Fig. 3B. Growth rates for ypk1D and ypk1D fpk1mutants should be quantified to support the conclusion that ypk1D fpk1 grows faster.

We performed growth curves for the wild type, *ypk1*Δ single mutants, and *ypk1*Δ *fpk1*Δ double mutants, and determined the growth rates.The results are shown in the new supplemental Fig. S2 and the text in line 181 has been modified accordingly..

Fig. 3C should be quantified (even though the results looks convincing, this would strengthen the conclusion).

The signals of the relevant bands have been quantified by densitometry, as explained in the methods section, and the differences between wild type and mutants are stated in the text (lines 191-193 and 198-199).

Figure 7. Ypk1 bands should be quantified.

The signals of theYpk1 bands have been quantified by densitometry and the differences between wild type and *pkh1*Δ and *pkh3*Δ mutants are stated in the text (lines 290-294).

Minor:

Figure 1 should be labeled with the genotypes in addition to strain numbers to make it less confusing.

Relevant genotypes have been added in the figure; the differences between the various *ypk1*Δ mutants are explained in the text (lines 119-135).
